# The contribution of Cyprus to non-communicable diseases and biomedical research from 2002 to 2013: implications for evidence-based health policy

**DOI:** 10.1186/s12961-018-0355-4

**Published:** 2018-08-17

**Authors:** Elena Pallari, Grant Lewison, Chryso Th. Pallari, George Samoutis, Mursheda Begum, Richard Sullivan

**Affiliations:** 1 King’s College London, Institute of Psychiatry, Psychology & Neuroscience (IoPPN), Centre for Implementation Science, Health Service and Population Research Department, David Goldberg Centre, De Crespigny Park, Denmark Hill, London, United Kingdom; 2 King’s College London, Kings Health Partners Comprehensive Cancer Centre, King’s College London, Institute of Cancer Policy, Guy’s Hospital, Great Maze Pond, London, United Kingdom; 3 King’s College London, Institute of Pharmaceutical Sciences, Faculty of Life Sciences & Medicine, Franklin-Wilkins Building, 150 Stamford Street, London, United Kingdom; 4 Department of Biological Sciences, University of Cyprus, P.O. Box 20537, 1678 Nicosia, Cyprus; 5 Centre for Primary Care and Population Health, St George’s, University of London Medical School at University of Nicosia, 21 Ilia Papakyriakou Street, Engomi, P.O. Box 24005, 1700 Nicosia, Cyprus

**Keywords:** Biomedical research, Non-communicable diseases, Cyprus, Clinical guidelines, Policy documents, Newspapers, Funding

## Abstract

**Background:**

Non-communicable diseases (NCDs) are the leading causes of disease burden and mortality at the European level and in Cyprus. This research was conducted to map the research activities of Cypriot institutions in five NCDs, namely oncology, cardiovascular diseases, diabetes, mental health and respiratory conditions.

**Methods:**

For the period 2002–2013, research in Cyprus was assessed on its biomedical outputs and compared to the rest of Europe relative to their GDP. The research output in the five NCDs was obtained and contrasted to their respective disease burdens. The results from each of the five NCDs showed the amount of cross-country collaboration with other researchers from other European countries and from the rest of the world, and the research level of the papers on a clinical to basic scale. For each NCD field the research application was assessed, whereas for oncology the research type was also assessed. Information was collected on the development of clinical guidelines, on Cypriot newspapers reporting on medical and policy documents and advisory committees’ output as well as research and funding organisations available in Cyprus, for potential evaluation of impact in health policy on the five NCDs.

**Results:**

Cypriot biomedical research output appeared appropriate in volume compared with its wealth and the expected value from a regression line for other European countries. However, it was focused particularly on the molecular mechanisms of transmittable or hereditary diseases, rather than on the five NCDs. Cyprus performs well in palliative care, which receives funding from several local charities and other non-profit organisations. Cyprus has the highest relative burden from diabetes in Europe, but the subject is largely neglected by researchers. Similarly, it suffers more from mental disorders than most of the rest of Europe, but the amount of research is relatively small. Respiratory conditions research is under-funded and under-researched too.

**Conclusions:**

The biomedical research portfolio in Cyprus is adequate in volume, but not well fitted to its pattern of disease. The means whereby research can be used to improve healthcare in the country are also unsatisfactory, although the Ministry of Health is now developing a comprehensive plan which will include the development of clinical guidelines and proposals for the evaluation of how healthcare is delivered on the island.

**Electronic supplementary material:**

The online version of this article (10.1186/s12961-018-0355-4) contains supplementary material, which is available to authorized users.

## Background

### Biomedical and non-communicable diseases (NCDs) research

Biomedical or medical research is conducted to improve healthcare [1] and involves basic, clinical, applied or translational research to provide better treatments to patients [2]. NCDs are chronic diseases attributable to modifiable risk factors such as lifestyle and nutrition behaviour, genetic, physiological or environmental factors [3]. There are four main NCDs according to WHO [3, 4], namely cardiovascular diseases (CVDs; including heart attacks and stroke), cancer, chronic respiratory diseases (including chronic obstructive pulmonary disease and asthma) and diabetes. Mental disorders are also considered a public health concern and are addressed on the WHO Global action plan as complementary to its work on the prevention and control of the other four NCDs [4].

### Cypriot healthcare system setting

**Fig. 1:**
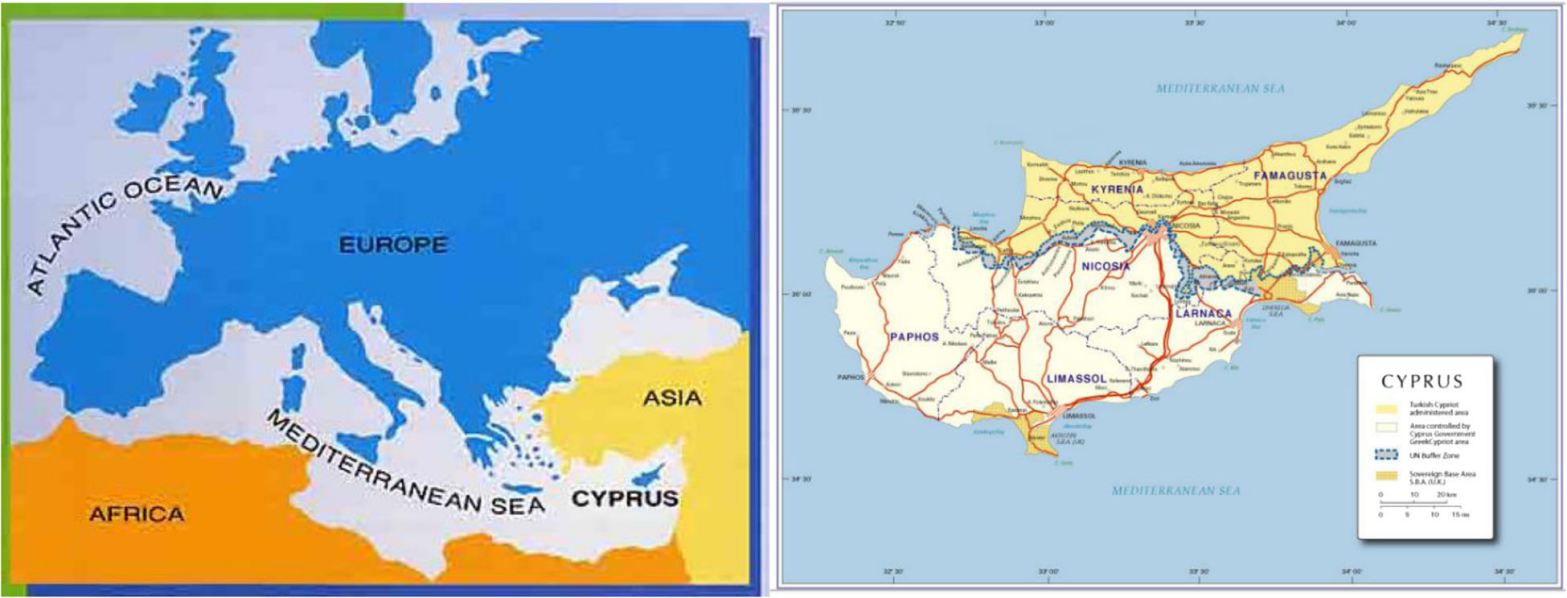
Map of Cyprus (adapted) [131, 132]

Cyprus is an island of the Eastern Mediterranean and a European Union Member State since 2004 [5]. Officially the Republic of Cyprus, it gained independence from the United Kingdom in 1960 and also became a member of the United Nations in that year [6]. Since the Turkish invasion in 1974, the island has been divided into two parts; the northern part under Turkish occupation, which is not recognised internationally, and the southern Greek-Cypriot part [6, 7]. Therefore, this paper only covers the situation in the southern part of the island which is government controlled, including all provided statistics and universities adjusted to this extent [7, 8]. The map of Cyprus is shown in Fig. 1. The government-controlled or southern part of Cyprus has five main districts [9], namely Nicosia (the capital, also known as Lefkosia), Famagusta (also known as Ammochostos), Larnaca, Limassol (also known as Lemesos) and Paphos, with a total population of 848,300 in 2015 [10].

Cyprus is the only Member State in the European Union without a universal National Health System [5, 11–14]. Since Cyprus joined the European Union over 12 years ago, the economic system underwent major systemic reforms and fluctuations, including restrictions from the European Central Bank (commonly known as Troika) to control healthcare expenditure [14–19]. The total health expenditure in Cyprus is below that of most other European Union Member States, at an average of 6.2% of gross domestic product (GDP) in 2013, with public expenditure at 2.8% of GDP and private at 3.4% [20, 21]. Private expenditures on the other hand, mainly consist of direct payments for private-sector healthcare services, some statutory co-payments, and premiums for private health insurance schemes, which are currently voluntary [11, 13–23]. Thus, the system is regressive but is accepted because most Cypriots prefer to use the private sector [16–18].

There are 11 main government-funded (public) hospitals in Cyprus across the five districts, 25 medical centres covering remote areas, and 74 private hospitals [24, 25]. The main challenges faced by the Cypriot health system include the increase in obesity, a high incidence of smoking and an unsatisfactory diet for many citizens [5], as well as the need to provide healthcare to immigrants and other at-risk populations such as conscripts and ethnic minorities [20–23]. In 2008, approximately 90% of all mortality in Cyprus was attributed to NCDs with CVDs accounting 43%, cancer 19%, diabetes 7% and respiratory diseases 6% [26]. The statistics of disease burden, in terms of the disability-adjusted life years (DALYs) for the world, the 31 European countries (the 28 European Union Member State and three Free Trade Member States: Iceland, Switzerland and Norway, collectively denoted as EUR31) and Cyprus are shown in Table 1.

**Table 1:** The disease burden (population and percentage values in thousands DALYs) for the world, the 31 European (EUR31) countries and Cyprus (WHO, 2012) [27]

		World		EUR31		Cyprus	
		DALYs (000’s)	%	DALYs (000’s)	%	DALYs (000’s)	%
All causes		2,735,162	100	155,304	100	216.1	100
Group 1	Communicable, maternal, perinatal and nutritional conditions	924,686	33.8	7548	4.9	8.1	3.7
Group 2	Non-communicable diseases	1,505,907	55.1	133,904	86.2	184.8	85.5
	Malignant neoplasms	222,567	8.1	30,228	19.5	35.1	16.2
	Trachea, bronchus, lung cancers	38,307	1.40	6611	4.26	7.2	3.34
	Breast cancer	17,644	0.65	2598	1.67	4.2	1.96
	Colon and rectum cancers	17,370	0.64	3492	2.25	2.9	1.34
	Lymphomas, multiple myeloma	9053	0.33	1355	0.87	2.1	0.96
	Prostate cancer	5812	0.21	1420	0.91	2.0	0.91
	Diabetes mellitus	58,810	2.2	4114	2.6	10.6	4.9
	Mental and behavioural disorders	182,841	6.7	20,352	13.1	31	14.4
	Unipolar depressive disorders	76,173	2.78	6428	4.14	11.6	5.37
	Alcohol use disorders	31,998	1.17	3965	2.55	7.3	3.38
	Alzheimer’s disease and other dementias	18,129	0.66	4780	3.08	4.7	2.17
	Anxiety disorders	27,389	1.00	2561	1.65	3.8	1.78
	Drug use disorders	15,120	0.55	1388	0.89	2.0	0.92
	Schizophrenia	14,033	0.51	1230	0.79	1.8	0.82
	Cardiovascular diseases	392,430	14.3	32,500	20.9	41.7	19.3
	Ischaemic heart disease	165,223	6.04	13,882	8.94	20.7	9.57
	Stroke	140,783	5.15	7942	5.11	6.9	3.21
	Respiratory diseases	136,698	5.0	7983	5.1	11.1	5.1
	Chronic obstructive pulmonary disease	92,118	3.37	4763	3.07	4.0	1.85
	Asthma	25,114	0.92	1570	1.01	2.8	1.30
	Other non-communicable diseases	512,560	18.7	38,726	24.9	55	25.5
Group 3	Injuries	304,570	11.1	13,853	8.9	23.2	10.7

Between 2000 and 2015, based on WHO data (data not shown), the burden from Group 1 diseases (communicable diseases) in Cyprus continued to decline (from 5.7% to 4.8%) while that from Group 2 diseases (non-communicable diseases) increased correspondingly, especially from cancer (from 16.4% to 19%) and mental disorders (from 8.9% to 11.2%). However, the burden from CVDs declined from 24.4% to 20.8%. There was also a decline in DALYs from Group 3 (injuries) from 10.3% to 8.0%, and notably in road traffic accidents (from 3.5% to 2.1%). These changes need to be considered when the appropriateness of the Cypriot medical research portfolio is being evaluated.

### Aim and study importance

The aim of this research paper is to examine five main questions about biomedical research in Cyprus:

- Is Cyprus doing enough biomedical research relative to its wealth?
- Is its portfolio appropriately designed to address the main diseases in Cyprus?
- Is the research emphasis attributed to the five NCDs appropriate to the burden affecting the Cypriot population?
- What is the research in terms of citations and international collaborations of Cypriot institutions?
- What impact is this research having on the Cypriot healthcare system and are there means to improve it?

We sought to examine the output of Cypriot research papers with particular focus on biomedical research and research on five NCDs, to highlight areas of prominence and to draw attention to research gaps. We also sought to examine the research institutions and funding organisations involved and make recommendations on how Cypriot biomedical research could better serve the health of the country. No study has previously been conducted to evaluate the biomedical research activities in Cyprus or to assess their impact on public health provision.

## Methods

### Research objectives

We examined the impact of Cypriot research output in five ways. Firstly, biomedical research was assessed relative to other researchers from Europe and the rest of the world in comparison to GDP, funding and research organisations in Cyprus. Additionally, biomedical research output was compared to the disease burden and its average annual growth was assessed on a percentage basis. Secondly, the research was examined for the five NCDs in terms of research level and cross-country collaboration. Further, the amount of research output was compared to the disease burden on each NCD and for individual diseases and, for each NCD, the various sub-disease applications were assessed, but only the research types for oncology were examined due to resource restrictions. Thirdly, clinical guideline (CG) development efforts on the island were evaluated. Fourthly, medical research reported in newspaper stories and its impact on influencing public opinion was assessed. Finally, policy documents and advisory committees’ output (relative to GDP and collaboration with other countries) on the five NCDs in Cyprus were evaluated to examine how health priorities are met.

### Data sources

The main source of data for this study was the Web of Science^©^ (WoS) Clarivate Analytics. The details of the papers were identified by means of special filters (proprietary of Evaluametrics Ltd.), downloaded to files and analysed by means of several Visual Basic Application (VBA) programmes (developed by Dr Philip Roe of Evaluametrics Ltd. see Acknowledgments) that identified the countries with which Cyprus collaborated and the subject areas of the individual papers. Outputs were compared to the Cypriot disease burden, as provided by WHO data. The performance of the leading Cypriot research institutions was determined based on citations from the WoS and on European clinical guidelines and newspaper stories. The sources of funding for Cypriot NCD research from 2009 to 2013 were also analysed from the data in the WoS. Finally, a small systematic literature review was conducted on the topic using the search statement TS = (Cyprus or Cypriot) to yield results (title, abstract and keywords) using the Science Citation Index on the WoS. All studies concerning any of the five NCDs directly affecting or in relation to the Cypriot population were selected and form part of the discussion section (no other synthesis or meta-analysis was performed as outside the scope of this study).

### Biomedical research output

The data were extracted, under a licence, from the WoS and were limited to articles and reviews from the 12 years, 2002–2013. The papers were selected by means of ‘filters’ whose precision and recall were determined by means of subject experts marking sets of papers as relevant, or not, from both the Science Citation Index Expanded and the Social Sciences Citation Index. There is some overlap between the two, and so papers appearing only in the latter were marked. The biomedical research papers were selected by means of a filter based on addresses and those on any of the five NCDs were selected by means of a set of filters based on specialist journal names and title words. Their bibliographic details were then downloaded from the WoS as a series of spreadsheets and processed by means of a special VBA programme. The data were then compiled in five Excel spreadsheets for analysis. Papers with an address in one of the cities or research institutions in North Cyprus were manually removed from the analysis, as the WoS does not distinguish it from South Cyprus.

### Country performance comparison

This information was corrected to reflect the true GDP for Cyprus through data mining (and prediction of Northern Cyprus GDP for 2010). Further VBA programmes allowed the addresses on each paper to be parsed to give the fractional count of Cyprus and other countries and show the amount of international collaboration. For example, a paper with one Cypriot and two United Kingdom addresses would be classified as Cyprus = 0.33 and United Kingdom = 0.67. This was particularly useful for the comparison of the country’s disease burden with its research output as it indicated whether it was relatively low or high. Additionally, the presented results show the annual growth of the Cypriot research output in the five NCDs and biomedical research overall.

### Funding organisations

It was also established to be worthwhile to explore the relationship between the number of funding bodies and citation counts and list the funding organisations in Cyprus, as there is no study in the literature on this. This is important in revealing if some NCD areas of research are under-funded compared to others, and if these outputs appear low in relation to need as well as which funders may have a bigger role to play in improving the situation. The 2009 cut-off date is the first year for which full funding information is available on the WoS, only available from the Science Citation Index database, hence our analysis of NCDs funding was done for papers from 2009 onwards. The average number of funding bodies per paper was also examined with respect to the disease area and type of research. The names of the funders are not standardised in the WoS and therefore they were coded to indicate not only their individual identity but also their sector (e.g. government, private-non-profit, industrial). The precision of the filters was better than their recall estimate, but this was not corrected for individual shortfalls in papers or funders by NCD. Instead, a 10% error was added in estimating the number of papers in each NCD to allow for more accurate prediction for the filters having higher precision (specificity) than recall (sensitivity). The total funding allocation was then calculated based on the fractional contribution of Cypriot institutions to each paper multiplied by the research paper cost. Then, the overall funding was divided by 5 to estimate the annual funding allocation for research for each funding organisation during this 5-year period. The methodology on the process of identifying acknowledgements of papers, funding coding and analysis is described elsewhere [27].

### Research institutions

Codes were also given to identify the fractional contributions of the leading Cypriot research organisations to each paper and any others on the island. A measurement of research contribution in the five NCDs was determined on a fractional count basis by examining the addresses of each of the NCD papers. For example, as above, if a paper in oncology was co-authored between a leading Cypriot institution and two United Kingdom institutions then, according to their addresses, that institution would score 0.33 for Cyprus and 0.67 for the United Kingdom (noted as foreign contribution). To be able to do this, each NCD paper was examined for at least a distinct Cypriot organisation on the address with a code assigned to each one. These institution codes were then converted with the help of a macro (see Acknowledgements) to determine the involvement of each of the identified Cypriot research centres to each paper in all five NCDs. This process enabled the percentage estimation of contribution, based on the total number of published papers involving Cyprus and the degree of influence on the research conduct in each of the NCDs.

### NCD research

Each of the five NCDs was examined in detail with the following codes assigned to:

- Cancer research (oncology): ONCOL
- Cardiovascular research, including stroke: CARDI
- Diabetes research: DIABE
- Mental disorders research: MENTH
- Respiratory disease research: RESPI

### Disease burden

For each NCD, data were also obtained on the burden of disease in Cyprus by WHO [28] to be compared with the research output.

### Research level of papers and journals of cited papers

From the paper title, a macro was applied to determine if the paper could be classified as ‘clinical’ or ‘basic’ or ‘both’, according to the presence of one or more words on two lists [29]. The research level of the journal in which the paper was published was also determined from a master list, based on the same scheme; clinical journals were classed as RL = 1 and basic ones as RL = 4, and ones in between were given an RL value as a decimal number between 1.0 and 4.0. These RL values were determined for groups of 5 years, 2000–2004, 2005–2009 and 2010–2013.

#### NCD research application

Each NCD paper was sub-classified according to their subject area of application, i.e. the disease or disorder that they were addressing. This was accomplished by means of ‘sub-filters’, developed in close consultation with one or more experts in each NCD. They consisted of sets of title words, and sometimes also of journal name strings, and for each NCD they were combined into a special macro so that the file of papers could be analysed very quickly. For example, a paper in oncology was coded ONCOL, on colorectal cancer would be coded as COL, while on CVD or stroke this would be coded as CARDI, and so on. Not all the papers in an NCD could be classed in this way as an application might not be indicated, meaning that the final tally is less than 100%, although some papers involved the study of more than one disease area or disorder. For this purpose, fractional counts were not used for the classification of papers by disease area. Instead, the estimation of the contribution to each type of research was done on an integer basis, so if a paper focused on both breast and ovarian cancer, then it would be classified under each category.

### Cancer research type

The main analyses of each paper were regarding the type of application or sub-disease in each of the NCDs, and for ONCOL, the type of research was also included (as sub-filters were developed only for oncology research). A similar system was used as the one described for the NCD research application to classify the ONCOL papers by research type such as genetics, surgery, etc.

### Clinical guidelines (CGs)

The evaluation for the existence of CGs in Cyprus was performed through an assessment of the websites of the Ministry of Health (MoH) [30] and the National Health Insurance Organisation in Cyprus [31]. Then, for each cited paper, the same methodology as described for the research paper output would be followed if the CGs provided scientific references that could be downloaded from the WoS, processed with macros and analysed further.

### Newspapers in Cyprus

The electronic archive for several newspapers was examined, e.g. Φιλελεύθερος (Phileleftheros), Πολίτης (Politis), to check for access to articles for the period under study (2002–2013) [32–35]. A methodology was devised with keywords for each of the five NCDs in English and translated into Greek to obtain relevant articles reporting research studies in both Greek and English newspapers in Cyprus (Table 2). As the newspapers’ archive did not go back much in time, an attempt was made to contact the Press and Information Office (PIO) in Cyprus [36] to gain access to these articles. Additionally, Factiva^©^ Dow Jones was employed as a news press database of over 10,000 newspapers, with the potential to provide access to any newspaper. The details of the relevant stories were noted in an Excel database (date, subject notes), along with details of the cited papers to identify the cited paper on the WoS, and downloaded as a txt file. These were then further processed by a macro.

**Table 2:** The keyword search strategy for the newspaper archive in English and Greek

The newspaper search strategy in English				
ONCOL	CARDI	DIABE	MENTH	RESPI
(cancer or leukaemi* or melanoma* or lymphoma*) and (research* or study or scientists or expert*)	(heart or stroke or blood pressure or hypertension) and (research* or study or scientists or expert*)	diabet* and (research* or study or scientists or expert*)	(addict* OR ADHD OR alcoholi* OR Alzheimer’s OR anorexia OR anxiety OR bipolar OR bulimia OR dementia OR depression OR hyperactivity OR schizophrenia OR self-harm* OR suicide*) and (research* or study or scientists or expert*)	(asthma or COPD or chronic obstructive pulmonary disease or allergic rhinitis or cystic fibrosis or emphysema) and (research* or study or scientists or expert*)
(καρκίνος or καρκινικ* or λευχαιμία* or μελάνωμα* or λέμφωμα*) and (έρευνα* or μελέτη* or επιστήμονες* or ειδικός*)	(καρδι* or εγκεφαλικό or αρτηριακή πίεση or πίεση αίματος or υπέρταση) and (έρευνα* or μελέτη* or επιστήμονες* or ειδικός*)	(διαβήτης* or διαβητικ*) and (έρευνα* or μελέτη* or επιστήμονες* or ειδικός*)	(εξαρτ* or εθισμ* or ΔΕΠΥ or αλκοολικ* or Αλτσχάιμερ or ανορεξία or διπολική or βουλιμία or άνοια or κατάθλιψη or υπερκινητικότητα or σχιζοφρένια or αυτοτραυματισμός or αυτοκτονία*) and (έρευνα* or μελέτη* or επιστήμονες* or ειδικός*)	(άσθμα or ΧΑΠ or Χρόνια Αποφρακτική Πνευμονοπάθεια or αλλαργική ρινίτιδα or κυστική ίνωση or εμφύσημα) and (έρευνα* or μελέτη* or επιστήμονες* or ειδικός*)

### Policy documents and advisory committee members’ research portfolio

Our initial thoughts on this type of impact were that European Union Member State governments would devise their health policies on the basis of research evidence, and that policy statements would have some scientific references. As these documents were of strategic focus [37–39], with no cited research studies, our research attention was turned to experts whom governments might be receiving advice from, i.e. the members of the advisory committees, who could be expected to advise their governments from the standpoint of their own research experience and expertise. The aim here was two-fold; firstly, to see in which disease areas European Union Member State governments were receiving advice (and whether this reflected the disease burden of the country), and secondly, to see if the members of the advisory committees were linked to researchers in other European Union Member States. A list of members of these advisory committees was obtained and then the papers they had authored and also covered in the WoS for the 5 years of study (2009–2013) were identified and classified as articles or reviews. For some of the advisers, the city in which they worked was listed to reduce the risk of finding papers by their homonyms. Nevertheless, there remained many papers by homonyms in fields remote from biomedical research, and so the topic search facility in the WoS was used to remove these. When the papers had been downloaded to files and converted to an Excel spreadsheet, the journals in which they had been published were further examined. Ones that were clearly non-medical were also removed, and titles of the ones in related fields (such as psychology or biology) were checked to see if they were relevant. Further, papers in medical fields unconnected to the five NCDs, such as gynaecology and infectious diseases, were removed.

The next piece of analysis was more complex and involved a comparison of the papers in the combined spreadsheet with the ones in the five large files of research papers in NCDs that was created for the period 2002–2013. It was assumed that the advisory committee papers would have had an address in Europe, even though a few committee members had addresses in another European Union Member State. A VLOOKUP function was performed to identify which papers were in cancer, diabetes, etc. This allowed to see the balance of the expertise available to the European Union Member State governments. The function not only provided information on which papers were in each of the five NCDs, but also the sub-fields within them.

## Results

### Biomedical research output

The number of biomedical research papers produced from Cyprus in the period 2002–2013 was 2177. The final research paper output, following the removal of papers from Northern Cyprus, some duplicates and papers in the field of physics, was 1757. Further classification of these papers as to whether they were on any of the five NCDs or other diseases, showed that 87% were on infectious diseases, basic laboratory research using animals or molecules or advancements in the field of biological sciences and bioengineering. Therefore, for the study period 2002–2013 there were 468 research papers published from Cypriot research institutions spanning across the five NCDs (Table 3). Of those, just 10% represented research concerning the health of the Cypriot population (for the full paper details see section NCD research and references [40–92]). The fractional contribution of Cyprus is also provided on Table 3, along with the percentage contribution.

**Table 3:** The research papers’ output from Cyprus on the five NCDs, 2002–2013

NCD	Papers (*N*)	Papers (fract *N*)	Papers (fract %)
ONCOL	189	74.9	39.6
CARDI	182	81.6	44.8
DIABE	18	8.0	44.4
MENTH	67	37.4	55.9
RESPI	12	4.8	39.9
TOTAL	468	206.7	44.2

*CARDI* cardiovascular diseases research, *DIABE* diabetes, *MENTH* mental disorders, *ONCOL* oncology (cancer), *RESPI* respiratory diseases

### Country performance comparison

**Fig. 2:**
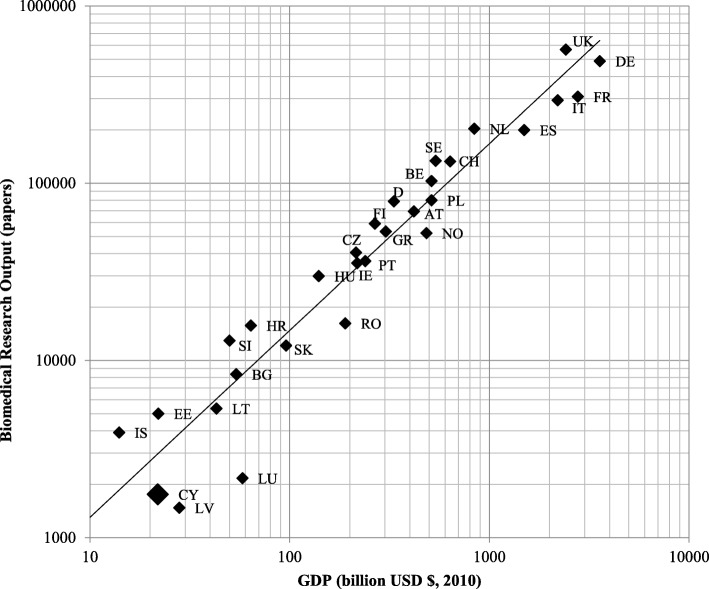
The countries contribution to biomedical research on a log-log scale from 2002 to 2013 in relative to its GDP in 2007

The GDP of Cyprus for 2010 was 23.13 billion USD, while that of Northern Cyprus is predicted at approximately 1.23 billion USD; therefore, the true GDP was estimated to be approximately 21.9 billion USD. GDP data were mapped for each EUR31 country against biomedical research output. This was to give an estimate of a country’s performance and allow comparison between countries based on their research activities. Figure 2 shows that Cyprus publishes approximately 40% below what would be expected (see large diamond in bold black on Fig. 2 below the trend line average). For example, Luxembourg, with twice as much GDP, is not performing any better, but Estonia with a similar GDP is producing twice more.

#### Disease burden and NCD research

**Fig. 3:**
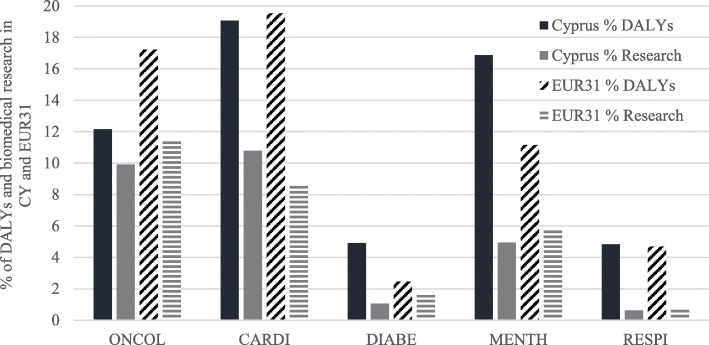
The percentage of disease burden (data were averaged for 2004 and 2012 to cover the study period) and biomedical research in Cyprus and the 31 European countries for the five NCDs

A comparison of the disease burden (data were averaged for 2004 and 2012 to cover the study period) with research outputs (percentage of all biomedical research) for Cyprus and Europe for the five NCDs (Fig. 3) showed that research in oncology is quite similar between Cyprus and the rest of Europe, although Europe has a higher cancer disease burden. Cyprus does more research on CVDs despite having a lower burden of disease, while the opposite is true in Europe. On diabetes and mental health, Cyprus has a higher burden of disease than the rest of Europe, yet it does very little research in these NCDs, while Europe performs approximately the same research relative to its disease burden. For respiratory conditions, the burden of disease is similar for both Cyprus and the rest of Europe, however, they both do little research.

### Average annual percentage growth of biomedical and NCD research output

The average annual percentage growth of the research output for biomedical research and each of the five NCDs was also calculated (Table 4). The highest is for oncology (21.2%), followed by biomedicine (18.5%) and CVDs (18.27%), and mental health (15%). On diabetes, there is a gap in research output between 2002 and 2007 and 2007–2010. On respiratory conditions, only publications from 2006 and 2010–2013 were retrieved.

**Table 4:** The average annual percentage growth (AAPG, %) of research output for Cyprus for the various NCDs (actual number of research papers provided)

Year/NCD	BIOMED	ONCOL	CARDI	DIABE	MENTH	RESPI
2002	53	4	5	2	3	0
2003	46	5	9	0	2	0
2004	58	7	4	0	5	0
2005	74	6	11	0	3	0
2006	83	8	8	0	2	2
2007	88	7	12	2	3	0
2008	133	12	10	0	3	0
2009	184	20	19	0	3	0
2010	221	23	23	1	7	2
2011	220	22	28	3	9	2
2012	256	39	28	5	10	4
2013	326	36	38	5	17	2
AAPG (%)	18.5	21.2	18.27	–	15.08	–

*BIOMED* biomedical, *CARDI* cardiovascular diseases research, *DIABE* diabetes, *MENTH* mental disorders, *ONCOL* oncology (cancer), *RESPI* respiratory diseases

### Research institutions

In total, there were 56 different organisations in Cyprus identified as conducting research, of which codes were assigned for each across the 468 NCD papers. Overall, there are six leading Cypriot research organisations, others focusing on research on either of the five NCDs, some other smaller institutions as well as foreign contributions from institutions abroad. Table 5 summarises the key findings. For the full contribution for each NCD by other institutions, see Additional file 1 (results for which are not shown on Table 5).

**Table 5:** The contribution (fractional counts of papers) to NCD research by the six leading research organisations in Cyprus, others in Cyprus, and foreign institutions

Institutions/NCD	ONCOL	CARDI	DIABE	MENTH	RESPI	TOTAL
UCY	14.4	16.1	0.5	14.2	0.0	45.1
CUT	10.3	10.4	1.0	5.9	2.2	29.8
NGH	7.4	16.9	0.0	0.0	0.3	24.6
CIN	7.8	13.2	1.3	0.2	0.0	22.5
BOC	16.8	0.8	0.0	0.0	0.0	17.5
UNN	2.0	0.4	0.7	6.0	0.2	9.3
FOREIGN	114.1	100.4	10.0	30.6	7.2	262.3
Other CY institutions	16.3	23.9	4.4	10.2	2.1	56.9
Total	189	182	18	67	12	468
Main six CY institutions %	30.0	25.4	23.8	38.8	26.6	29.9
Foreign %	60.4	55.2	55.6	45.6	60.1	56.0
Other CY %	9.7	19.5	20.6	15.5	13.3	14.1

*BOC* Bank of Cyprus Oncology Centre, *CARDI* cardiovascular diseases research, *CIN* Cyprus Institute of Neurology & Genetics, *CUT* Cyprus University of Technology, *DIABE* diabetes research, *Other CY* other Cypriot research organisations on the island, *MENTH* mental disorders research, *NGH* Nicosia General Hospital, *ONCOL* oncology (cancer) research, *RESPI* respiratory diseases research, *UCY* University of Cyprus, *UNN* University of Nicosia

In oncology research, in terms of fractional contribution to the NCD papers, the main research institutions are the University of Cyprus, the Bank of Cyprus Oncology Centre (BOCOC), the Cyprus Institute of Technology, the Cyprus Institute of Neurology & Genetics, and the Nicosia General Hospital.

On cardiovascular research, the main institutions are Nicosia General Hospital, the University of Cyprus and the CING, while other organisations include the BOCOC, the University of Nicosia (previously known as Intercollege), and the Vascular Screening and Diagnostic Centre in Nicosia.

There are three main institutions accounting for diabetes research, namely the University of Nicosia, Nicosia General Hospital and the University of Cyprus. Other contributors include the European University, Ygeia Polyclinic and seven other Cypriot institutions.

On mental health research, the University of Cyprus, the University of Nicosia and the Cyprus Institute of Technology conduct most research, while the rest comes from the European University, Ministry of Agriculture, the Research and Education Foundation of Child Health in Nicosia, Frederick University, Open University, Archbishop Makarios III Hospital, Cyprus Anti-Cancer Society, and Kenthea (non-profit, non-governmental organisation (NGO)).

Although minimal research (less than 3% of NCD research) is conducted on respiratory conditions, this comes from the Cyprus Institute of Technology and the Cardiovascular Disease Educational and Research Trust (CCDERT), as well as from other Cypriot institutions like the Archbishop Makarios III Hospital, Nicosia General Hospital and the Research and Education Foundation of Child Health in Nicosia.

### Funding organisations

There are currently three types of Cypriot organisations that fund medical research on the island, namely government agencies or departments, private non-profit organisations and industrial companies. Overall, we identified 15 bodies funding research in the five NCDs, as shown in Table 6. The funding information was available for 310 out of the 468 papers to which Cypriot researchers contributed to for 2009–2013 in the five NCDs. Biomedical research conducted in Europe from 2009 to 2013 was estimated to cost an average of €260 k per published paper [93]. For Cyprus, this cost, based on its GDP (see trend on Fig. 2) was estimated to be marginally lower, at approximately €200 k per published paper. For the contribution of these different funding organisations to the Cypriot NCD research output and the amount spent on research per year, based on the fractional contribution of Cypriot institutions to these papers, see Table 6. The breakdown of the papers that obtained specific funding (2009 onwards, over 66% of NCD research output in Cyprus had funding information available), shows that 34% is on CARDI, 30% on ONCOL, 20% on MENTH, 9% on DIABE and 7% on RESPI from Cypriot institutions.

**Table 6:** The 15 main funding bodies, their contribution and expenditure for NCD research in Cyprus between 2009 and 2013

Type of funding organisation	Contribution (fractional counts)	Expenditure (€m/year)	Name of funding bodies
Government	28.8	1.3	
Agencies	24.3	1.1	Cyprus Cardiovascular Disease Educational and Research Trust (CCDERT)
			Electricity Authority of Cyprus (AHK)
Departments	4.5	0.2	Government of the Republic of Cyprus, including the Ministry of Health and the other 10 Ministries
Private-non-profit	20	0.9	
Charities	2.4	0.1	Anti-Cancer Society
			Cyprus Heart Foundation
			Cyprus Telethon
Foundations	3.7	0.2	AG Leventis Foundation
			Cyprus Research Promotion Foundation
			Marfin Popular Bank
Academic	9.2	0.4	Cyprus University of Technology
			University of Cyprus
Other non-profit	4.8	0.2	Child Health Institute
			Cyprus Institute of Neurology & Genetics
			Cyprus Nurses and Midwives Association
Industry	2	0.1	Private companies, e.g. Chemical Block Ltd.

According to data provided from the Cyprus Volunteer Commissioner’s charity office, there are 5400 NGOs in Cyprus interacting with them directly or indirectly. The role of the charity volunteers and NGO Commissioner is to organise fundraising events and cooperate with universities and researchers on organising activities, seminars or workshops, but not to provide funding. The charity does not give grants for research projects and may have discounted the tax exemptions mainly to land property issues, customs duty or tax exemptions on their income. NGOs can be subsidised depending on the action of Government Services of the European Projects. In government grants and schemes, the conditions are notified by each service, and in European projects, their evaluation is carried out as specified in the application guide.

### NCD research

#### Disease burden

The NCD disease burden in Cyprus for 2012 [28] for the top 12 sub-diseases causing more than 1% of DALYs, compared with the analysed research output (papers) published by researchers in Cyprus, is shown in Table 7.

**Table 7:** The disease burden (DALYs, %) in Cyprus from 2012 data from WHO [30] and the research output (papers, %) for the various non-communicable diseases (NCDs) and their sub-diseases

NCD sub-diseases	DALYs, 2012 (%)	Research (papers), 2002–13 (%)
Ischaemic heart disease	9.6	4.1
Unipolar depressive disorders	5.4	5.0
Diabetes	4.9	16
Alcohol use disorders	3.4	1.3
Trachea, bronchus, lung cancers	3.3	1.4
Stroke	3.2	22.9
Alzheimer’s disease and other dementias	2.2	4.1
Breast cancer	2.0	16.2
Chronic obstructive pulmonary disease	1.9	0.0
Anxiety disorders	1.8	5.3
Hypertensive heart disease	1.5	2.6
Colon and rectum cancers	1.3	4.2

### Cross-country collaboration

**Fig. 4:**
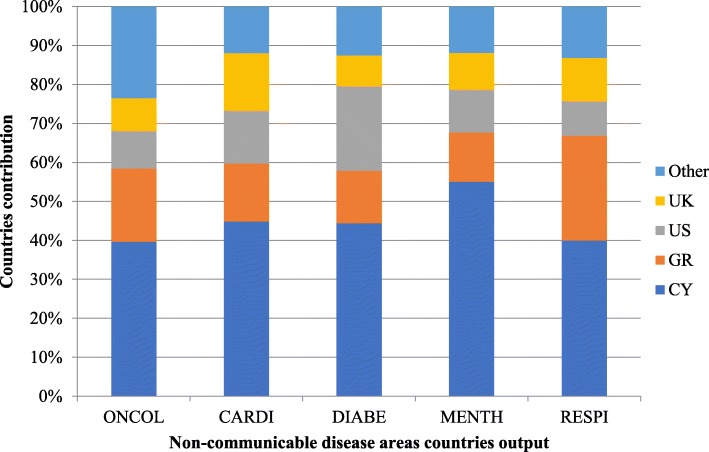
The percentage of countries’ contribution to research papers output in each of the five NCDs. Index: *CY* Cyprus, *GR *Greece, *US* United States, *UK* United Kingdom, *Other* countries from the rest of the world

The greatest contribution to Cypriot research papers comes from researchers based in Cyprus, followed by researchers from Greece, the United States and the United Kingdom, who provide the largest contributions to these papers (from foreign institutions). For each NCD, these countries’ order is different, based on their percentage contribution (Fig. 4).

### Research level of papers and journals of cited papers

**Fig. 5:**
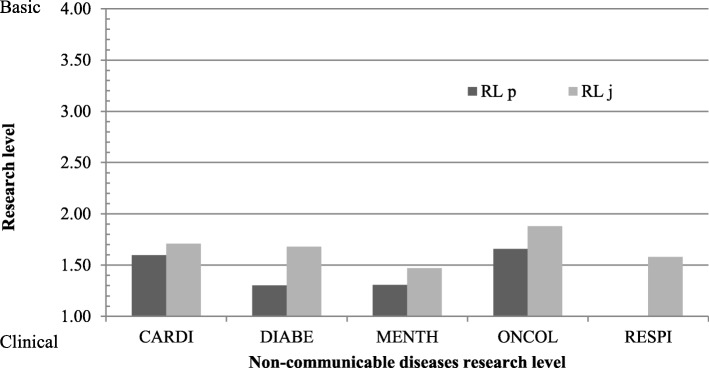
The research level of the papers (RL p) and the journals in which they are cited (RL j) in each of the five NCDs

The research level of papers and their respective journals in which they were published was assessed in each of the five NCDs. Overall, NCD research papers from Cyprus tend to be more clinical, while the journals these are published in appear to be more basic (Fig. 5). For oncology papers, the overall research level is more basic while, for mental disorders, it is the most clinical amongst the five NCDs.

### NCD research application

#### Oncology research application

**Fig. 6:**
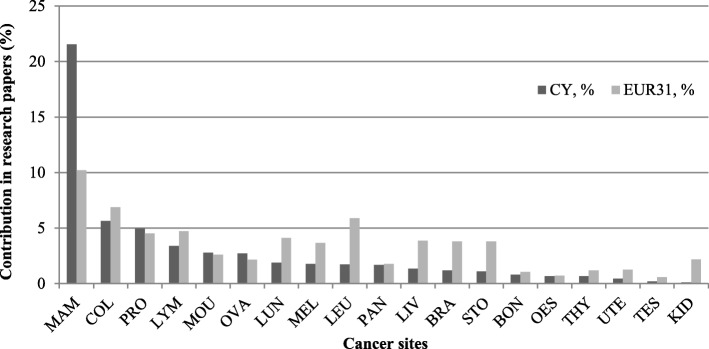
The percentage of oncology studies’ output in Cypriot and EUR31 cancer research papers. Oncology (cancer type) index: *MAM* breast, *COL* colon/rectum, *PRO* prostate, *LYM* lymphoma, *MOU* mouth (head & neck) cancer, *OVA* ovaries, *LUN* lung, trachea, bronchus, *MEL* melanoma (skin), *LEU* leukaemia (blood), *PAN* pancreas, *LIV* liver, *BRA* brain, *STO* stomach, *BON* bone, *OES* oesophagus, *THY* thyroid, *UTE* uterus, *TES* testicles, *KID* kidney

The 189 research papers in oncology were mainly attributed to breast cancer (21%), followed by colorectal (8%), prostate (5%), lymph node carcinoma (3%), brain and ovarian cancer (2% each) and various other cancer site manifestations (14% in total) (Fig. 6).

### Cancer research type

**Fig. 7:**
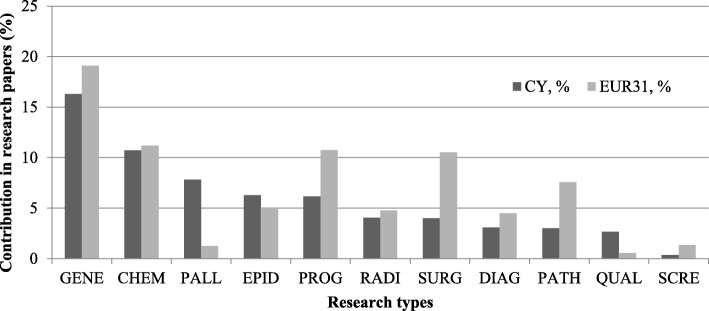
The percentage of oncology studies on different research types for Cyprus and EUR31 countries. Oncology research type: *GENE* genetics, *CHEM* chemotherapy, *PALL* palliative care, *EPID* epidemiology, *PROG* prognosis, *RADI* radiotherapy, *SURG* surgery, *DIAG* diagnosis, *PATH* pathology, *QUAL* quality of life, *SCRE* screening

Analysis by the type of research in the various cancer sites showed that genetics received the greatest attention (16%), followed by chemotherapy (14%), prognosis (9.5%), radiotherapy (7%) and other (22% in total) (Fig. 7).

#### Cardiovascular diseases

**Fig. 8:**
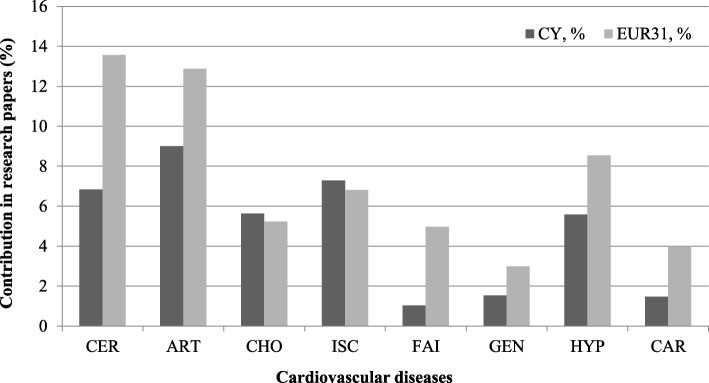
The percentage of Cypriot and EUR31 cardiovascular papers contribution in each of the disease areas. Cardiovascular diseases index: *CER* cerebrovascular disease, *ART* arterial disease, *CHO* hypercholesterolemia, *ISC* ischemic heart disease, *FAI* heart failure, *GEN* genetic heart disease, *HYP* hypertension, *CAR* cardiomyopathies

There were nine disease areas identified from the Cypriot cardiovascular research papers (Fig. 8). The main category is arrhythmias and conduction disorders, including atrial fibrillation (18%), followed by research on arterial diseases, including atherosclerosis, especially aortic aneurisms (12.6%). Next, was coronary heart disease, including acute myocardial infarction and angina (9%), cerebrovascular disorders including stroke (8.8%) and hypertension (6.6%). The remaining are heart valve diseases, including chronic rheumatic diseases, heart failure and cardiomyopathies (6%).

#### Diabetes research

**Fig. 9:**
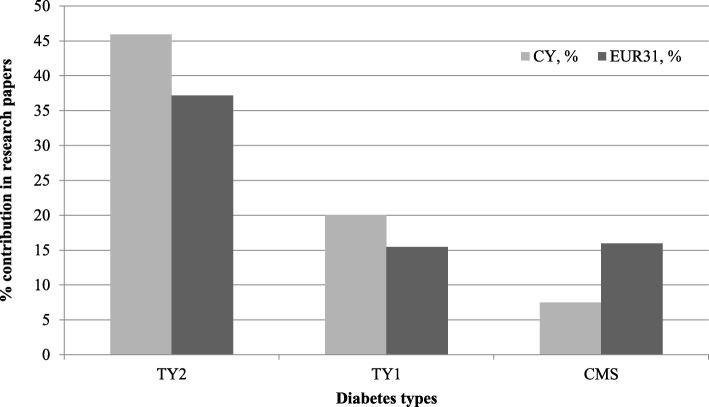
The percentage of the Cypriot diabetes research papers in each of the sub-disease types in comparison to the 31 European countries. Diabetes manifestations index: *TY1* type 1 diabetes mellitus, *TY2* type 2 diabetes mellitus, *CMS* cardiovascular complications (cardiometabolic syndrome)

Almost half of the Cypriot research papers on diabetes are attributed to type 2 diabetes (45.6%) (Fig. 9). The other types of diabetes included metabolic syndrome (11.1%), type 1 (11.1%), maturity onset diabetes of the young (5.6%) and neonatal diabetes (5.6%), accounting for 33.3% all combined.

The percentages of all DALYs for each of the EUR31 are listed in Table 1. On the diabetes disease burden specifically, there is a large variation between countries, from 4.6% for Cyprus, the largest in EUR31, to 1.2% for the United Kingdom, the smallest. It is curious that despite having the lowest percentage disease burden from diabetes, the United Kingdom nevertheless publishes substantially more than would be expected and easily the most papers of any of the EUR31 countries. In contrast, Cyprus publishes very little. The Mediterranean and southern European countries appear to suffer from this disease the most, with the notable exception of Latvia.

#### Mental health disorders

**Fig. 10:**
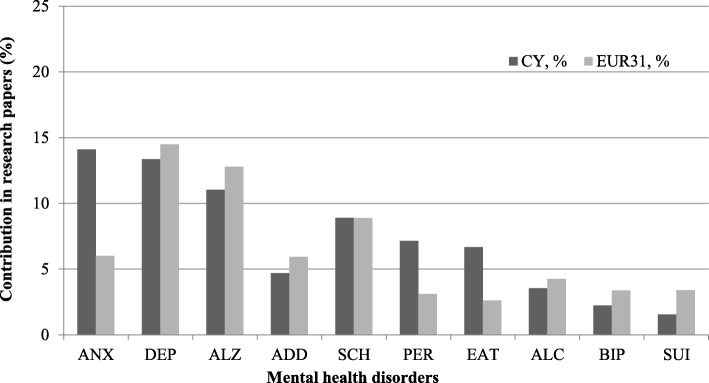
The percentage of the Cypriot and EUR31 mental health research papers in each of the sub-disease types. Mental health disorders index: *ANX* anxiety disorder, *DEP* unipolar depression, *ALZ* Alzheimer’s and other dementias, *ADD* drug use and other addictions, *SCH* schizophrenia, *PER* personality disorder, *EAT* eating disorder, *ALC* alcohol abuse, *BIP* bipolar affective disorder, *SUI* suicide and self-harm

There were 10 mental health sub-disorders (Fig. 10), with the main one being anxiety (16%). Depression was the second largest area of mental health research (10%), Alzheimer’s and other dementias, and personality disorder accounting for 8.8% each, addiction (7.4%), schizophrenia (6%), eating disorders (4%), alcohol abuse, bipolar affective disorder, and suicide and self-harm research accounting for 3% each, and attention-deficit hyperactivity (1.5%).

#### Respiratory conditions

**Fig. 11:**
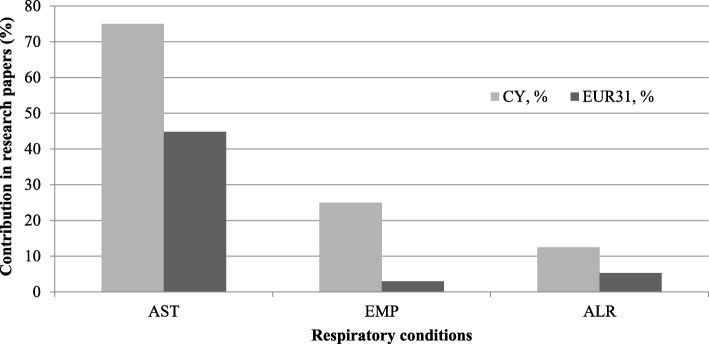
The percentage of the Cypriot and EUR31 respiratory research papers in each of the condition’s subtypes. Respiratory conditions index: *AST* asthma, *EMP* emphysema, *ALR* (chronic) allergies, e.g. hayfever

From the respiratory research of Cypriot researchers, it is evident that the greatest emphasis is on asthma (75%), while allergies and emphysema account for less than 10% each (Fig. 11). Additionally, there is no research performed on COPD despite accounting for 1.9% of all Cypriot DALYs.

### Clinical guidelines (CGs)

We found that, currently in Cyprus, there have been some efforts made around the development and implementation of CGs, notably on primary healthcare [94], secondary/tertiary healthcare [95] and laboratory exams [96]. To date, the only three CGs relevant to the five NCDs (CARDI, DIABE and MENTH) are in the format of PowerPoint presentations specifically on hypertension, type 2 diabetes and depression, which are available only in the Greek language without any reference to research studies. Therefore, no further processing or analysing of these guidelines was undertaken.

### Newspapers in Cyprus

The only electronic archive accessible for any newspaper in Cyprus was through Factiva^©^ Dow Jones database for *Cyprus Mail*, an English-language newspaper circulated in Cyprus. The database provided results only for the time period 2008–2013, as it could not go back further in time (personal communication with PIO) [97]. All the other newspapers, both Greek and English language, in Cyprus had much smaller time coverage through their electronic archive (up to a year) and these were neither available to be accessed upon subscription nor through Factiva. However, the digital archive provided by the PIO, dates back to 2007. For earlier articles, only manual retrieval of the relevant articles was possible by scanning each and every single paper ever published on a microfilm; an impossible task or at least so for the purpose of this project. Through Factiva, there were 11 stories in the *Cyprus Mail* reporting research on any of the five NCDs out of the 396 total hits using the developed keywords (Table 2). These 11 articles were identified on the WoS and their details were downloaded for further processing. Only four articles came from Cypriot researchers (whether in collaboration or not with other institutions from abroad) and hence no further analysis was done.

### Policy documents and advisory committee members’ research portfolio

Following a meeting with the Chief Medical Officer at the MoH, the message was that there are several strategic plans published by the MoH relevant to NCD research priorities, namely oncology [37], diabetes [38] and dementias [39] (mental health) publicly available from the MoH website in the Greek language. In a personal communication letter, the focus on strengthening the research-base on cancer in particular was made clear, with the main strategy structured around four pillars [37] as follows. The first pillar is on the prevention of cancer and provision of screening of population programmes, with emphasis on sustainability in the long-term. The second pillar is on diagnosis and treatment, particularly on government support for the upgrade of facilities of the BOCOC and tertiary research centres in collaboration with the University of Cyprus Medical School and the set-up of clinical expertise specifically in breast and colon cancer care. The third pillar is on the development of CGs on palliative care around community support and establishment of palliative care centres like the BOCOC and the Limassol General Hospital Oncology Unit. The final pillar concerns the development of proper infrastructure around the co-ordination of all the centres to excel in the provision of cancer care services, including potential collaborations with overseas experts.

Only health advisory committees relevant to the five NCDs were analysed. From the Cypriot MoH website [97], the following organisations were individually searched, and the lists of members were obtained either directly from the website or from contact with the organisation’s administration:

- Cyprus Anti-Drugs Council
- Cyprus Mental Health Commission
- Health Insurance Organisation
- National Bioethics Committee
- Patients’ Rights Commission

The work on the advisory committees’ assessment in five non-communicable diseases for Cyprus and other European countries is presented in another research work [98]. The results for Cyprus show that only three out of the five committees currently operating in Cyprus are involved in public health policy-making. These are the Cyprus Anti-Drugs Council, the Cyprus Mental Health Commission and the National Bioethics Committee for Biomedical Research, with 26 committee members in total and 36 produced papers during 2002 and 2013. The papers were mostly in communicable diseases, hereditary disorders and genetics, with only four being on diabetes and one on mental health. However, all 36 papers were analysed based on the addresses and showed that 51.1% stem from Cyprus, while the remaining is split between Europe (38.1%) and the rest of the world (10.8%).

## Discussion

The overall research activities of researchers from Cypriot institutions show that, in biomedical research, the focus is on infectious conditions, hereditary disorders and mutations, research involving biological systems engineering, medical physics, and genetics or molecular biology research, with minimal concentration on NCDs (Table 3).

### Biomedical research output

#### Country performance

The main health concerns in Cyprus are attributed to the healthcare issues of an ageing population such as the increase in healthcare costs associated with an increase in long-term chronic degenerative diseases and an increase in cancer and CVDs [5, 17]. Yet, the biomedical research output of Cyprus shows that researchers are producing approximately 60% of the research that would be expected relative to the country’s GDP. For example, when compared with European Union Member States having a 2.5-times higher GDP performance, such as Luxembourg, Cyprus is performing better in terms of research output, but not compared to Lithuania which has a 1.6-times lower GDP (also below the regression line shown in Fig. 2). Perhaps there is an association between public healthcare expenditure and focus on biomedical research for Cyprus, as reflected from the results.

#### Disease burden and NCD research

Although the total research on the five NCDs as a percentage is less than half of the disease burden, the situation in Cyprus is not dissimilar from EUR31, except for cancer, for which Cypriots have a higher disease burden (has increased from 2004) yet perform less research on. The notable exception here is breast cancer, which is over-researched eight-fold compared to its disease burden (Table 7) and two-fold compared to EUR31 ouput (Fig. 6).

#### Average annual percentage growth

Overall, the Cypriot research output for biomedical research represented 2.5% of the total European output in 2002–2013. However, although Cyprus is slightly below average on its output performance relative to its GDP, the Cypriot research output is rapidly increasing, indicating the pace at which biomedical and cancer research on the island is being conducted (Table 4). It is of interest that, despite the high incidence of diabetes on the island, there is a mismatch of research, evident from the lack of publications. The two areas which are particularly under-researched are diabetes and respiratory conditions.

#### Research institutions

There were 56 institutions identified as conducting research in Cyprus. Overall, the specific percentage contributions attributed to each NCD research type shows that there are five main institutions conducting 30% of research output in Cyprus in any of the five NCDs (Table 5). These are the University of Cyprus, Cyprus Institute of Technology, Nicosia General Hospital, CING, and BOCOC. To our knowledge, this is the first time an effort is made to establish this information and perhaps recognise the research input made by these organisations on public health research. However, the extent to which these institutions play a role in influencing the research portfolio and funding allocation is not clear. Perhaps the Cypriot government should recognise the research contributions from these institutions by providing more funding support for NCD research. The remaining 14% stems from other institutions on the island, while 56% is from foreign contributions. For a full list of these institutions, see supplementary material in Additional file 1.

#### Funding organisations

Based on the available funding information, ONCOL and CARDI receive the greatest amount of financial support relative to the other NCDs. This also shows the rapid increase (see average annual percentage growth in Table 4) between 2009 and 2013, from which we can only predict that the output is expanding. For these papers, the main funding support is from the Cypriot Government followed by the not-for-profit sector (Table 6). There was no pharmaceutical sponsorship on any of these papers, and currently the manufacturing companies on the island do not appear to financially support any biomedical research. This finding, in combination with the disease burden, indicates that the funding landscape should therefore change priorities towards NCDs. That said, disease-specific charities like the Anti-Cancer Society may be needed to support more basic oncology research. Furthermore, there are no hospital trusts contributing to NCD research either.

### Non-profit, NGOs in Cyprus

There were three NGOs funding research in the five NCDs in Cyprus, namely the Child Health Institute, Cyprus Institute of Neurology & Genetics and the Cyprus Nurses and Midwives Association (Table 6). However, there are several others that do not appear to fund research, although these are known in the medical sphere in Cyprus. These include the Cyprus Society of Medical Informatics, a non-profit, NGO chartered in 1997 [99], the MedicAlert Foundation registered as a volunteering organisation in 1975 [100], the Medical Association of Cyprus [101], The Cyprus Pharmacists Organization [102], the Cyprus Diabetics Association [103] and Vagoni Agapis [104]. There are also various medical charities, three of which support NCD research in Cyprus; the Cyprus Anti-Cancer Society established in 1971 [105], Cyprus Heart Foundation [106] and the Cyprus Telethon [107]. There are also 11 other charities supporting patients in need, namely the Cyprus Association of Cancer Patients and Friends established in 1986 [108], the Friends For Life [109], Ronald McDonald House Charities Cyprus [110], Cyprus League For Chest Diseases [111], Evie Sofianou Foundation [112], Elpida Foundation For Children With Cancer & Leukemia [113], Ayios Nektarios Charity Foundation [114], Cyprus Samaritans [115], the Cancer Charity Shop [116], the Karaiskakio foundation [117] and the Cyprus Red Cross Society founded in 1950 as a branch of the British Red Cross [118]. Other charities in Cyprus include the Friends for Life Limassol Hospice [119], The Friends’ Hospice Paphos [120], Nicosia General Hospital Oncology Unit [121], Limassol General Hospital Oncology Unit [122] and the BOCOC [123].

### Government agencies

In Cyprus, there is a main government agency identified to support cardiovascular research, the Cyprus Cardiovascular Disease Educational and Research Trust [124]. Additionally, the findings show that the Electricity Authority of Cyprus [125] supports biomedical research and this is compatible with previous research showing that semi-state organisations are part of the healthcare delivery sub-system [17]. Additionally, this previous study demonstrated that the Government of the Republic of Cyprus, including the MoH and the other 10 Ministries, and several other private and government organisations, including the Fire Brigade and the Traffic Branch of the Police Force, administer various public health programmes to support this effort [17].

### NCD research

The importance of the findings from the biomedical research portfolio in Cyprus and NCDs research output are put into context from the literature which specifically focuses to the Cypriot population (see Methods section). The systematic search yielded 4134 studies regarding Cyprus or Cypriot in their title or abstract. Following screening of the title and abstracts, the relevant articles concerning research on any of the five NCDs are discussed in this section (*n* = 53; see references [40–92]).

### Disease burden

The NCD research represents only approximately 27%, which is less than half of what it should be compared to the 58% of DALYs affecting the Cypriot population (Fig. 3). This is particularly important when setting priorities in the research agenda, as this representation of NCDs from the whole of the biomedical research portfolio needs to be more balanced. Furthermore, there is also the need for the Cypriot government and other funders that fund research to shift support to specific sub-NCD research areas more appropriately.

### Cross-country collaboration

Cyprus has approximately 40% research contribution to its NCD output and up to a 55% dominant presence in its mental health research papers (Fig. 4). For the other countries, the highest contribution, accounting for almost a third (27%), is from Greece on respiratory conditions research papers, 22% from the United States on diabetes research, 15% on cardiovascular research from the United Kingdom and 24% from the rest of the world in oncology research. This cross-country collaboration could indicate that either Cypriot research institutions already have pre-existing networks with specific institutions within these countries or may even indicate a preference towards disease-specific research from these countries.

### Research level of papers and journals

Although the journals publishing the NCD research papers appear to be more basic than the papers themselves (Fig. 5), it is important to note these NCD papers, out of the whole biomedical portfolio, are meant to be more clinical and applied in nature, and predominantly exclude very basic laboratory research.

#### Oncology research

Cypriot research focuses predominantly on breast cancer within oncology (about a fifth) and less on areas like lung cancer (Fig. 6), mainly in terms of susceptibility and screening. Additionally, the greatest oncology research type was on genetics (Fig. 7). Breast cancer poses a problem across Cyprus, especially in certain geographical areas, with higher rates of mortality in urban than rural/mountainous areas, as well as in places of high population density [40]. Strategic efforts to tackle this include the formation of the Europa Donna Cyprus Forum, a non-profit charitable organisation, which has demonstrated continued progress in raising awareness, contributing to research and encouraging screening in Cyprus [41, 42]. Cypriot women’s intentions to undergo their initial mammography screening are predicted by a range of psychological (e.g. self-efficacy, confidence in one’s ability to get screened) and sociodemographic factors (e.g. level of education) [43, 44]. This is important because many strong predictors of breast cancer have been reported amongst Cypriot women, including the age of menarche, breastfeeding and family history of breast cancer [45]. The latter risk factor has triggered explorations of the role and pathogenic mutations of cancer susceptibility genes, particularly related to genes *BRCA1* and *BRCA2*. Mutations of both genes are highly prevalent in Cypriot women with early-onset breast cancer [46, 47]. Subsequently, the DNA repair pathway has been explored, given that deficiencies in the pathway could lead to the genetic instability of cells (e.g. cell malfunctioning and tumorigenesis). Genetic variations in single-nucleotide-polymorphisms XRCC1 and XRCC2 are reported to influence breast cancer susceptibility in Cypriot women aged 40–70 years [48]. Given the greater role of the *BRCA2* gene in Cypriot familial breast cancer compared to the *BRCA1* gene [49, 50], papers have explored the associations between several single-nucleotide-polymorphisms in this gene and breast cancer susceptibility, demonstrating an increased risk amongst women carrying a specific variant of both the *BRCA2* and *MRE11A* genes [51]. However, women carrying *BRCA2* mutations show clinical courses with better prognoses than those carrying *BRCA1* mutations, in terms of a longer time to first relapse as well as fewer relapses, but both carriers showed higher survival rates than expected for the stage and grade of their cancers [47].

A striking finding is that research on palliative care (Fig. 7) conducted in Cyprus is a lot more (8%) than European research (1%). Although the provision of end-of-life services both in the private and public sector are limited, there are several institutions providing such facilities, including the BOCOC, Paphos General Hospital and Limassol General Hospital Oncology Unit. This finding is also supported from evidence by the MoH [37] (personal communication), which shows that the strategic focus in oncology was to strengthen this often-neglected type of provision of cancer care at the end of life. Other studies have also explored Cypriot health services and care for the general treatment and management of cancer. This is important since greater time in and familiarity with treatment is related to better quality of life amongst Cypriot cancer patients [52]. Other studies have shown that the main oncology centre in Cyprus, treating over 80% of cancer patients on the island, provides several multi-disciplinary occupational health clinic sessions with diverse consultations (e.g. vaccinations, smoking cessation, etc.) [53]. However, facility-based care is not always possible, and so finding effective home-care systems such as DITIS [54] and community-based palliative services for isolated rural areas [55] have been explored. This speciality is nonetheless underdeveloped in Cyprus in terms of policy, inclusion in national healthcare system and physician’s knowledge of prescribing opioids, but there has been some progress in these areas [56, 57]. Opioid consumption in Cyprus is low despite good availability and non-excessive regulatory restrictions, with physicians appearing to under-treat cancer pain. Less than 60% of patients in three studies across Cypriot cancer centres were prescribed some form of analgesia, opting mainly for paracetamol and non-steroidal anti-inflammatory drugs, with very few prescribed strong opioids [58]. There is, however, growing prescription of opioids over morphine, which could provide insight into the lowering fear and stigmatisation of opioids amongst patients and their families.

Tobacco smoking is widespread across Cyprus, and most individuals presenting with tobacco-related cancers compared to other cancers tend to be older (late 60s) and male rather than female in both rural and urban areas, although more so in the latter [59]. The highest rates of these cancers were found in certain geographical locations, namely Larnaca, Paphos and Lemesos, with variations in cancer types in each district (e.g. lung cancer incidence highest in Larnaca). Palliative care is available in cancer units within hospices, private clinics and home-care services across Cyprus, with two main charities promoting and providing community-based palliative care, namely Cyprus Anti-Cancer Association and PASYKAF [55, 57]. Other philanthropic sources of support and treatment are the Palliative Care Centre ‘Arodafnousa’, the Thalassemia International Federation, the Cyprus Red Cross, the Rehabilitation Centre ‘Melathron Agoniston for the National Organization of Cypriot Fighters (EOKA)’, the Cyprus Association of Cancer Patients and Friends, and the Centre for Preventive Paediatrics [17].

#### Cardiovascular research

Ischaemic heart disease was the leading cause of death in Cyprus, killing 1000 people in 2012 [60]; however, the findings show that research from Cypriot institutions is focused on stroke and less on hypertensive heart diseases (Fig. 8), although the latter has a very small disease burden (Table 7). Nevertheless, there might be a public health concern as metabolic syndrome incidence increases coronary artery disease, stroke and type 2 diabetes. In light of the aforementioned literature, Cypriot research has examined ways to assess CVDs and create effective interventions to reduce their burden. Studies have developed a data mining system to assess the risk of different cardiovascular heart events [61] and a verbal dyspnoea rating scale for measuring clinical symptoms in individuals with acute heart failure [62]. Research has also attempted to successfully apply a variety of Greek versions of assessment tools to Cypriot populations. This includes the Greek versions of the Multidimensional Scale of Perceived Social Support for people with heart failure [62]. Social support is an important factor to consider in patients with CVD [63] and many studies have assessed its relationship to adherence to exercise recommendations amongst heart failure patients, although further evidence is needed here [64]. The Greek version of the Acute Coronary Response Index for people with acute coronary syndromes has also been applied to a Cypriot population [65], in which the beliefs, attitudes and knowledge of patients with the condition influences whether early treatment is sought in Cyprus [66]. Knowledge seems to be key [63] not only in seeking early treatment [64], but also after undergoing interventions such as cardiac surgery for both the patient and their relative [67]. Other Greek versions of measures applied to Cypriot populations include the Montreal Cognitive Assessment to assess mild cognitive impairments in coronary artery disease [68] and the Delirium Observation Scale to assess delirium, a neuropsychiatric syndrome, in cardiac patients [69].

The impact of a variety of risk factors for CVD pertaining to Cyprus has been explored, especially amongst the elderly population. Research has addressed many environmental and lifestyle factors. For example, a relationship between high temperatures in Cyprus and risks of cardiovascular mortality, particularly for ischaemic heart diseases, has been reported [70]. Such risks appear greatest on the actual days of high temperatures and risks remain elevated the following day, which is concerning given Cyprus is situated in a warm region, which may worsen with increased global warming. Additionally, lifestyle factors, such as diet, physical activity and smoking, play important roles in CVD risk [71] and conditions like acute coronary syndromes [82]. Long-term access to nutrition (e.g. dieticians) and advanced healthcare services (i.e. both primary and secondary services rather than just primary units) seem to be correlated with decreased burdens of common cardiovascular risk factors amongst the elderly Cypriot population [73]. However, after controlling for relevant lifestyle factors including diet, physical activity and smoking, psychological factors such as depression seem to play an important role, where individuals displaying high levels of depressive symptoms exhibit more CVD risk factors (e.g. hypertension) than those at the lower end of the spectrum [74].

Hypertension is a key CVD risk factor, in which mental health, notably anxiety and depression, commonly affect the quality of life of Cypriots with this condition, more so than physical health (e.g. daily activities and self-care) [75]. Multi-faceted interventions, grounded on several theories and models, have been developed in Cyprus to provide better quality of care for hypertensive patients in primary care settings [76]. Such approaches, like one incorporating both an electronic medical record system and guidelines for managing clinical diseases, showed positive outcomes for hypertensive patients on many measures including blood pressure, total cholesterol and patient satisfaction [77]. Whilst a majority of physicians in Cyprus are aware of and adhere to guidelines on managing hypertension, there is great scope to improve not only their awareness of specific European scientific guidelines (e.g. those published by the European Society of Hypertension due to their relevance to the Cypriot population), but also better treatment of low- and high-risk hypertensive patients through more accurate prescriptions of medication and better recording of clinical information (e.g. smoking behaviour, lipid values, etc.) [78, 79].

#### Diabetes research

The prevalence of known diabetes in Cyprus is amongst the highest in Europe and the ratio of known to undiagnosed cases is 2:1 [80]. From the five NCDs under study, the burden of diabetes is the greatest amongst the rest of Europe (EUR31 countries) and Cypriot researchers within the field primarily focused on type 2 diabetes mellitus (Fig. 9). This research focus can be justified as diabetes is in line with the presence of metabolic syndrome in adults [81] and its increasing prevalence due to diets containing high levels of processed meat (e.g. barbeques and kebabs), smoking habits and a lack of exercise.

Two studies were conducted in the Cypriot population, on type 2 diabetes and impaired glucose tolerance, namely using a Hypoglycaemia Perspectives Questionnaire [80] and an oral glucose tolerance test [81], respectively. The first study was conducted on patient samples in Cyprus and in the United States to assess the validity of Hypoglycaemia Perspectives Questionnaire as a tool to capture the attitudes towards hypoglycaemia of affected individuals within the community [80]. The results showed that Cyprus had more males, fewer obese subjects and greater adherence to oral medication or insulin compared to the United States, as well as fewer reported hypoglycaemic events within a week [80]. The second study, conducted solely on 1200 subjects in Cyprus [81], showed a very high prevalence of diabetes and impaired glucose tolerance, indicating the need for interventions aimed at impaired glucose tolerance and the metabolic syndrome. The findings confirm those of a previous cross-sectional study. This study showed a substantial increase in the prevalence of obesity and overweight children and adolescents in Cyprus within 10 years, especially for those residing in rural areas [82], indicating the need for better interventions targeting early detection of type 2 diabetes, especially for obese children residing in remote parts of the island. Another study on type 1 diabetes mellitus in the Cypriot population showed a rising incidence of 4.44 per 100,000 person-years within a 10-year period [83]. These findings need to be taken into account in designing prevention strategies for the population, as well as more research on better management of the condition considering its rise.

#### Mental health

It is argued that 1 in 5 Cypriots suffer from a mental health disorder [17]. We found that research on mental health in Cyprus is primarily on anxiety and depression (Fig. 10). Suicide and self-harm research account only for 3% and evidence shows that these are neglected research topics. However, a transferrable lesson to be learnt perhaps by other medical fields in Cyprus, or even other countries, is that despite being a relatively small country, Cyprus concentrates a lot more on applied clinical research than basic. This is reflected by the development and provision of services that serve the needs of mentally ill patients. The mental health services in Cyprus underwent reconfiguration under the Psychiatric Care Reform, where the services on mental health provided mainly through the Athalassa Mental Hospital transferred to the community [20]. This de-institutionalisation included the development of the National Mental Health Centre for the extension of provision of services to children and adolescents, set-up of psychosocial rehabilitation programmes, clinical counselling and substance abuse therapy provided by the Therapeutic Unit for Addicted Persons and Anosis therapeutic units [17].

From the systematic literature search, research on mental disorders affecting the Cypriot population predominantly focused on specific mental health issues concentrating on the impact on caregivers rather than on sufferers, with just a single paper each for suicide, schizophrenia, dementia, eating disorders and depression. The earliest paper assessed suicide rates amongst the Christian Cypriot population from 1988 to 1999, which was low in relation to Europe, fuelled mainly by mental health problems and represented a growing problem, especially for men as they age [84]. This is perhaps unsurprising given that many Cypriot families carry the burden of caring for family members with mental health problems, such as those with schizophrenia, displaying high levels of worry about their welfare and reporting greater supervision needs for those who are male and have dementia [126], and an increasingly older Cypriot population that has led to high levels of burden, clinical depression and poor quality of life amongst caregivers [85]. However, mental health research has reported poor mental health amongst people who formally provide care as a profession, with 10% of Greek-Cypriot mental health nurses showing symptoms of anxiety and depression related to emotional exhaustion and detached/alienated relationships with patients [86]. Other studies have focused specifically on the mental health of women. One study found that single mothers exhibited depression levels that are three times that of the general population, related to factors like financial constraints and single-motherhood status [87]. Another study assessed female Greek-Cypriot university students, reporting an association between disordered eating and many psychological variables including higher levels of anxiety about weight and internalisation of thin body ideals, findings that are consistent with other populations living in warmer climates [88].

#### Respiratory conditions

There is a very small amount of published research papers on respiratory conditions for 2002 to 2013 (Table 4). Although Cyprus suffers about the same as the rest of Europe, they each perform minimal research, and respiratory conditions are the NCDs with the least amount of research done to match its disease burden (Fig 3). We found that the literature that does exist, almost entirely focuses on asthma, particularly on epidemiological and environmental risk factors amongst younger populations, opting for similar methodological approaches of cross-sectional study designs and questionnaires (Fig. 11). Studies have explored asthma in relation to vitamin D levels, showing how they tend to be lower amongst asthmatic adolescents compared to their non-asthmatic counterparts, especially those with severe forms [89]. Another paper compared populations, finding that, whilst children and adolescents of the Greek-Cypriot community showed lower prevalence rates of wheezing and rhinoconjunctivitis, those from the Turkish-Cypriot community displayed many protective factors (e.g. rural living) but also had the highest exposure to tobacco smoke [90]. Two studies have assessed asthma in relation to air quality, with one reporting short-term changes in air pollution and dust storms being associated with an increased risk of hospitalisations due to respiratory causes [91], whilst the other assessed asthma and respiratory symptoms manifestation amongst Greek-Cypriot adolescents as a result of proximity to three power plants [92].

### Clinical guidelines (CGs)

There are at present no Cypriot CGs. The Cypriot healthcare system is lagging behind in terms of universal healthcare coverage and quality management of its healthcare services. The government of Cyprus has recently approved the introduction of a single payer National Health System, the long awaited National Health Insurance Scheme which will provide universal health coverage, unify the healthcare services in the public and private sector and establish the tools for quality management such as a universal information technology system, CGs, clinical audits, etc. [127]. However, it is intended by the Cyprus MoH to develop some CGs for the island based on those published in England, namely the National Institute for Health and Care Excellence (NICE). From various sources of written publication, notably news press articles on collaboration with the United Kingdom, it is evident that there are efforts by the government of the Republic of Cyprus, healthcare professionals, the Cyprus Medical Association, Cyprus Medical Council, Cyprus Scientific Societies, and other organisations to assemble a team of experts to adapt and adopt these guidelines to the Cypriot context [128, 129]. A national CG group has been formed and has already adapted CGs in specific diseases, i.e. diseases with high cost and high volume. Cyprus has accepted the guideline adaptation process rather than the de novo development of CGs, basically due to cost concerns and avoidance of time consuming development processes. The main challenges that have been identified during implementation of the first CGs in Cyprus healthcare is the acceptance by physicians, the monitoring of guidelines introduction, the patient involvement in the development process as well as the lack of an independent body responsible for overseeing the development and introduction of CGs.

### Newspapers

Newspapers provide an important way for the dissemination of medical information [130]. However, our search only generated 11 stories in the study period (only from 2008 onwards), four of which cited a paper with Cypriot authors, but six Cypriot papers were cited by stories from other countries. Although specific keywords were used to retrieve relevant articles in any of the five NCDs, only 2.7% of the total number of newspaper articles was relevant by reporting research. Furthermore, as the number of relevant stories reporting research in any of the five NCDs is extremely low, that meant that we could not yield any statistically significant results. The lack of research reportage in the Cypriot media, whether in Greek, Russian or English language, indicates a potential lack of awareness around issues that are important and are neglected by reporters which perhaps impacts the public interests. This could in turn influence funding or research agenda priorities. Such potential reporting bias or misrepresentation of major health issues should be further explored in a future study to identify any mishaps between research, funding spending and health policy-making, and perhaps a better communication between researchers and reporters.

### Policy documents and advisory committee members’ research portfolio

The focus of the Cypriot MoH on the strategy on cancer, diabetes and dementia is the first step in identifying research priorities on the public health policy agenda and of pointing funding gaps in these areas. However, the three health advisory committees in Cyprus were not primarily concerned with the five NCDs, and only six out of the 36 biomedical research papers published by their members were on a single NCD (DIABE). Therefore, as the findings are limited to these three sub-diseases, further attention is needed in the various cancer types, more specifically lung and colorectal cancer (to justify the research already done on breast cancer). Additionally, in liaison with the various non-profit NGO funding bodies and the medical schools recently established on the island, this is a very good starting point to shift attention to NCDs currently affecting the Cypriot population. Additionally, although there are three committees providing guidance on public health topics predominantly on mental health (another piece of evidence demonstrating that this NCD receives strong government support), there is still a gap of research focus in the other NCDs. The explored research portfolio of advisory committees’ experts demonstrated the lack of integrating clinical expertise and scientific evidence in research or health policy-making in any of the five NCDs, as only about one paper in 10 studies covers this topic.

## Conclusions

Following a series of healthcare reforms and policy reconfiguration around the support and provision of health services by the Republic of Cyprus’ government, there are many gaps in the way biomedical research is conducted, reported and implemented. In the last couple of years, important initiatives have been undertaken to introduce major reforms in the Cyprus healthcare system. For example, efforts towards introducing CGs, other quality improvement measures and strategic plans towards public health have been made. However, there is still a mismatch between research output as well as funding for the diseases afflicting the Cypriot population. Furthermore, there appears to be an imbalance between the amount of research in Cyprus and the rest of Europe on communicable and non-communicable diseases, in view of the latter imposing 85% of the total burden (in DALYs). This was borne out by the results, as research outputs in the five NCDs totalled only 28% of the European biomedical research output whereas the five NCDs together accounted for 55% of the total DALY burden. This suggests that some re-balancing of the overall biomedical research portfolio would be in order, although some biomedical research is rather basic and could yield long-term benefits for many different diseases. The largest research output by Cypriot researchers was on cancer, although the burden from CVD was 20% greater in 2012. Within cancer, breast cancer was the leading research and funding area, but this is disproportionate to its disease burden. Specifically, Cypriot researchers focus more on breast cancer rather than colorectal and lung cancers, which affect more of the Cypriot population. Similarly, in CVDs, stroke gets the most attention, while ischemic heart disease should be researched more. These efforts are supported by funding from various Cypriot government departments. Mental health appears to have an appropriate amount of funding and health service provision, while oncology attracts somewhat more than enough support, whereas respiratory conditions research is clearly under-funded. Additionally, the main aim of evaluating committee members’ publications was to see whether the advisers were well-connected to research in other European countries, or whether they were relatively lacking in international contacts, or better connected to United States or Greece than to the rest of Europe. This would then show if health policy in the Member States was informed by research in the European Research Area, so that lessons learned in one country could benefit others. However, no conclusions can be made for NCDs, as the main focus of their recommendations and research ouput has been on communicable diseases. This study can be used to inform the changes in health policy in Cyprus, which are now being planned, by an assessment of how research can contribute to the improvement of healthcare provision on the island.

## Additional file

Additional file 1: **Table S1.** Cypriot research institutions. (DOCX 33 kb)

## Abbreviations

BOCOC: Bank of Cyprus Oncology Centre
CARDI: cardiovascular diseases research
CCDERT: Cyprus Cardiovascular Disease Educational and Research Trust
CGs: clinical guidelines
CING: Cyprus Institute of Neurology & Genetics
CVD: cardiovascular diseases
DALYs: disability-adjusted life years
DIABE: diabetes
EUR31: 31 European countries
GDP: gross domestic product
IGT: impaired glucose tolerance
MENTH: mental disorders
MoH: Ministry of Health
NCDs: non-communicable diseases
NGO: non-governmental organisation
ONCOL: oncology (cancer)
PIO: Press and Information Office
RESPI: respiratory diseases
VBA: visual basic application
WoS: Web of Science
