# Supplementary material for: The contribution of Cyprus to non-communicable diseases and biomedical research from 2002 to 2013: implications for evidence-based health policy
Source: Health Res Policy Syst. 2018 Aug 17;16:82. doi: 10.1186/s12961-018-0355-4 (PMC6098664; doi:10.1186/s12961-018-0355-4)
Supplement: Supplementary file 1 — Table S1. Cypriot research institutions. (DOCX 33 kb) [file 12961_2018_355_MOESM1_ESM.docx]

# APPENDIX

## *Cypriot Research Institutions*

| **Code** | **CARDI** | **DIABE** | **MENTH** | **ONCOL** | **RESPI** | **TOTAL** | **Full institution’s code** |
| --- | --- | --- | --- | --- | --- | --- | --- |
| **AED** | 0.20 | 0.00 | 0.00 | 0.37 | 0.00 | 0.57 | Europa Donna Cyprus |
| **AGC** | 0.00 | 0.00 | 0.00 | 0.00 | 0.00 | 0.00 | American Genetics Centre |
| **AHI** | 0.65 | 0.50 | 0.00 | 0.75 | 0.00 | 1.90 | American Heart Institute |
| **AMH** | 0.33 | 0.00 | 0.48 | 0.50 | 0.45 | 1.76 | Archibishop Macarios III Hospital |
| **APH** | 0.25 | 0.00 | 0.00 | 0.34 | 0.00 | 0.59 | Apollon Private Hospital |
| **ARH** | 0.33 | 0.00 | 0.00 | 0.25 | 0.14 | 0.73 | Aretae Medical Centre |
| **ASH** | 0.33 | 0.00 | 0.00 | 0.00 | 0.00 | 0.33 | Asclepios Antiaging Centre |
| **ATM** | 1.15 | 0.70 | 0.00 | 1.26 | 0.00 | 3.11 | Ayios Therissos Medical Diagnostic Centre |
| **BOC** | 0.75 | 0.00 | 0.00 | 16.79 | 0.00 | 17.54 | Bank of Cyprus Oncology Centre |
| **CAS** | 0.00 | 0.00 | 0.50 | 1.07 | 0.00 | 1.57 | Cyprus Anti-Cancer Society |
| **CBC** | 0.00 | 0.00 | 0.00 | 0.45 | 0.00 | 0.45 | Chemical Block Company |
| **CCD** | 1.91 | 0.20 | 0.00 | 0.00 | 1.00 | 3.11 | Cyprus Cardiovasular Diseases Centre |
| **CED** | 0.00 | 0.00 | 0.00 | 0.00 | 0.00 | 0.00 | Centre of Endocrinology & Metabolism |
| **CHF** | 0.35 | 0.00 | 0.00 | 0.00 | 0.00 | 0.35 | Cyprus Heart Foundation |
| **CIB** | 0.42 | 0.00 | 0.00 | 0.17 | 0.00 | 0.58 | Cyprus Institute of Biomedical Science |
| **CIN** | 13.18 | 1.34 | 0.20 | 7.80 | 0.00 | 22.52 | Cyprus Institute of Neurology and Genetics |
| **CSH** | 0.00 | 0.00 | 0.00 | 0.29 | 0.00 | 0.29 | The Center for Study of Hematological Malignancies |
| **CTM** | 0.00 | 0.00 | 0.00 | 0.50 | 0.00 | 0.50 | Cognitive Behavioural Therapy Clinic |
| **CUT** | 10.44 | 0.98 | 5.87 | 10.30 | 2.19 | 29.78 | Cyprus University of Technology |
| **CYC** | 0.00 | 0.00 | 0.33 | 0.00 | 0.00 | 0.33 | Cyprus College of Business |
| **CYI** | 0.00 | 0.00 | 0.00 | 0.31 | 0.00 | 0.31 | Cyprus Institute |
| **DPC** | 0.05 | 0.00 | 0.00 | 0.00 | 0.00 | 0.05 | Nicosia Diabetes Centre (Endocrinology & Diabetes Clinic) |
| **EDC** | 0.33 | 0.00 | 0.00 | 0.00 | 0.00 | 0.33 | Alpha Evresis Diagnostic Center |
| **EIC** | 0.07 | 0.00 | 0.00 | 0.03 | 0.00 | 0.10 | EPOS-IASIS Centre |
| **EMC** | 0.00 | 0.00 | 0.00 | 1.00 | 0.00 | 1.00 | Evangelistria Medical Center in Nicosia |
| **EPH** | 0.00 | 0.00 | 0.00 | 0.33 | 0.00 | 0.33 | Evangelismos Private Hospital in Paphos |
| **EUU** | 0.17 | 0.30 | 2.12 | 0.33 | 0.00 | 2.92 | European University of Cyprus |
| **FOR** | 100.39 | 10.01 | 30.57 | 114.07 | 7.21 | 262.25 | Foreign institutions |
| **FRU** | 1.90 | 0.00 | 1.00 | 0.00 | 0.00 | 2.90 | Frederick University |
| **INC** | 2.54 | 0.00 | 1.25 | 0.00 | 0.00 | 3.79 | University of Nicosia (previously known as Intercollege) |
| **IPH** | 0.00 | 0.00 | 0.00 | 0.33 | 0.00 | 0.33 | Hippocrateon Private Hospital |
| **KAD** | 0.00 | 0.14 | 0.00 | 0.00 | 0.14 | 0.29 | Cyprus Social & Economic Research Centre at CUT |
| **KCC** | 0.00 | 0.00 | 0.00 | 0.33 | 0.00 | 0.33 | Kes College in Nicosia |
| **KKF** | 1.20 | 0.00 | 0.00 | 0.00 | 0.00 | 1.20 | Karaiskakio Foundation |
| **KNO** | 0.00 | 0.00 | 0.34 | 0.00 | 0.00 | 0.34 | KENTHEA. Center for Education about Drugs and Treatment of Drug Addicted Persons |
| **LGH** | 0.53 | 0.00 | 0.25 | 1.63 | 0.00 | 2.41 | Limassol General Hospital |
| **MBC** | 0.00 | 0.00 | 0.00 | 0.13 | 0.00 | 0.13 | Mendel Center for Biomedical Sciences |
| **MOA** | 0.33 | 0.00 | 0.50 | 0.17 | 0.00 | 1.00 | Ministry of Agriculture |
| **MOF** | 0.20 | 0.00 | 0.00 | 0.00 | 0.00 | 0.20 | Ministry of Foreign Affairs |
| **MOH** | 0.67 | 0.00 | 0.00 | 0.08 | 0.00 | 0.75 | Ministry of Health |
| **MOL** | 0.50 | 0.00 | 0.00 | 0.00 | 0.00 | 0.50 | Ministry of Labour, Welfare and Social Insurance |
| **MSC** | 0.20 | 0.00 | 0.00 | 0.00 | 0.00 | 0.20 | MEDSONIC Ltd Company |
| **NGH** | 16.89 | 0.00 | 0.00 | 7.39 | 0.33 | 24.61 | Nicosia General Hospital |
| **NUP** | 0.00 | 0.00 | 0.50 | 0.00 | 0.00 | 0.50 | Neapolis University |
| **OPU** | 0.33 | 0.00 | 0.83 | 0.83 | 0.00 | 1.99 | Open University (Healthcare Management Department) |
| **OTH** | 3.63 | 0.64 | 1.00 | 1.73 | 0.00 | 7.00 | Other smaller CY organisations |
| **PGH** | 1.00 | 0.00 | 0.00 | 0.00 | 0.00 | 1.00 | Paphos General Hospital |
| **REC** | 0.40 | 0.00 | 1.10 | 0.00 | 0.20 | 1.70 | Research and Education Institute of Child Health |
| **SGH** | 0.14 | 0.00 | 0.00 | 0.00 | 0.00 | 0.14 | Larnaca General Hospital |
| **TIF** | 0.00 | 0.00 | 0.00 | 0.00 | 0.00 | 0.00 | Thalassemia International Federation |
| **TSH** | 0.33 | 0.00 | 0.00 | 0.00 | 0.00 | 0.33 | Timios Stavros Hospital |
| **UCY** | 16.09 | 0.50 | 14.17 | 14.37 | 0.00 | 45.13 | University of Cyprus |
| **UIC** | 0.00 | 0.00 | 0.00 | 0.44 | 0.00 | 0.44 | Cyprus Association of Cancer Patients and Friends |
| **UNN** | 0.39 | 0.73 | 5.98 | 1.96 | 0.20 | 9.26 | University of Nicosia |
| **VSD** | 3.16 | 1.95 | 0.00 | 0.00 | 0.00 | 5.11 | Vascular Screening and Diagnostic Centre |
| **YGP** | 0.25 | 0.00 | 0.00 | 1.58 | 0.00 | 1.83 | Ygia Polyclinic |
| **YHL** | 0.00 | 0.00 | 0.00 | 1.12 | 0.00 | 1.12 | Yasoo Health Ltd |
| **Total** | 182.00 | 18.00 | 67.00 | 189.00 | 11.86 | 467.85 | n/a |
| **Main %** | 25.36 | 23.78 | 38.84 | 29.97 | 26.58 | 29.87 | n/a |
| **Foreign %** | 55.16 | 55.62 | 45.63 | 60.35 | 60.07 | 56.04 | n/a |
| **Other %** | 19.47 | 20.60 | 15.53 | 9.67 | 13.35 | 14.09 | n/a |

n/a: Non-applicable
